# Use of Herbal Medicines Among Breastfeeding Mothers in Tanzania: A Cross-Sectional Study

**DOI:** 10.3389/fphar.2022.751129

**Published:** 2022-04-28

**Authors:** Valeria Phelician Millinga, Hyea Bin Im, Jung Hye Hwang, Soo Jeung Choi, Dongwoon Han

**Affiliations:** ^1^ Department of Global Health and Development, Graduate School, Hanyang University, Seoul, South Korea; ^2^ Institute of Health Services Management, Hanyang University, Seoul, South Korea; ^3^ Department of Obstetrics and Gynecology, College of Medicine, Hanyang University, Seoul, South Korea; ^4^ Department of Preventive Medicine, College of Medicine, Hanyang University, Seoul, South Korea

**Keywords:** herbal medicine, breastfeeding, health behavior, tanzania, cross-sectional study

## Abstract

**Background:** There are limited data on the use of herbal medicines (HM) among breastfeeding mothers, despite the fact that knowledge of the possible benefits or harms of HM use has a direct relationship with the health of infants, especially in resource-limited countries. The study aims to determine the prevalence and factors associated with HM use among breastfeeding mothers in Tanzania.

**Methods:** The study followed a cross-sectional design using a structured survey questionnaire. Survey participants were recruited from the reproductive and child health clinic at Uhuru health center in Morogoro, Tanzania. The survey instrument comprised of 34 questions, including demographic information, the pattern of HM use during breastfeeding, and women’s perceptions of HM. Chi-square test and logistic regression were used for data analysis using SPSS ver. 24.0.

**Results:** The majority of the respondents (53.8%) used HM during breastfeeding. The most commonly used HM was black pepper (*Piper nigrum* L.; 80.0%), followed by pumpkin seeds (*Cucurbita pepo* L.; 18.0%). About one-third (27.5%) of HM users discussed their use with their healthcare providers. In addition, higher education levels and low breastmilk supply were identified as potential predictors of HM use.

**Conclusion:** The practice of HM use among breastfeeding mothers in Tanzania is popular to ease breastfeeding difficulties. However, the issue of the safety or effectiveness of HM is still an unknown agenda. This awakens the need to evaluate HM’s safety, efficacy, and quality through pharmacological studies for scientific evidence. Lastly, a clinical guideline should be developed in healthcare settings to promote open dialogues between the healthcare providers and mothers to ensure the safe use of HM.

## Introduction

Globally, there is an increasing number of studies on women’s lifelong use of herbal medicines (HM). Several studies have documented women’s HM use in various life stages, starting from menstruation, pregnancy, childbirth, postpartum, and menopause (Ngoma and Siachapa, 2017; Wang J. et al., 2018; Moini Jazani et al., 2018; Zheng et al., 2019; Adane et al., 2020; Sumankuuro et al., 2020). Especially during some of these life stages, such as pregnancy, delivery, and the postpartum period, women’s HM use should be paid close attention to, as seemingly naive HM that has no negative impact on non-pregnant women can have a detrimental effect on the child and pregnant woman (Budzynska et al., 2012; Ahmed et al., 2017).

According to the World Health Organization, exclusive breastfeeding (EBF) is an essential practice during the postpartum period for maternal and child health (WHO, 2015), as it is one of the most effective ways to promote both mothers’ and newborns’ physiological and psychological health (Ebina and Kashiwakura, 2012; Antonakou et al., 2013; Al Sabati and Mousa, 2019; Mikšić et al., 2020). Breastmilk is more natural and cost-effective than bottle feeding and ensures ideal nutrition for newborns, thus promoting infant health, providing essential bioactive nutrients, and lowering the risks for various infections and hospitalizations (Oguchi et al., 1997; Butte et al., 2002; Hauck et al., 2011; Ajetunmobi et al., 2015). In addition, breastfeeding facilitates mother and child bonding by promoting physical intimacy between the mother and child (Johnson, 2013; Liu et al., 2013). However, up to 92% of nursing women encounter various difficulties during their breastfeeding journeys (Wagner et al., 2013). Some mothers seek an alternative mode of care, such as HM, to alleviate the problems due to the lack of access to conventional healthcare services and the belief that HM is more natural and safe to use than conventional medicine (Augustino and Gillah, 2005; Sim et al., 2013; Aleandri et al., 2014; Zheng et al., 2020). Some of the commonly used HM among breastfeeding mothers include fenugreek (*Trigonella foenum-graecum* L.), ginger (*Zingiber officinale* Roscoe), fennel (*Foeniculum vulgare* Mill.), shatavari (*Asparagus racemosus* Willd.), and aniseed (*Pimpinella anisum* L.). Moreover, various phytochemical compounds (e.g., glycosides, alkaloids, flavonoids, and minerals) found in medicinal plants modulate hormone levels and act as lactogenic agents (Bnouham, 2010; Dandotiya et al., 2013; Sim et al., 2013).

Nevertheless, there is much debate about whether the existing evidence related to breastfeeding mother’s HM use supports their safety and efficacy (Sim et al., 2013; Bettiol et al., 2018a) as previous studies show inconclusive evidence explaining the mechanism behind herbs as galactagogues, requiring further research to determine HM’s clinical efficacy (Anderson, 2017; Bowman, 2017; Wang S. et al., 2018; Sibeko et al., 2021). Furthermore, inappropriate use of certain HM during breastfeeding was found to cause allergic reactions and neurotoxic effects on newborns (Bnouham, 2010). Yet, irrespective of the lack of scientific evidence and potential harm to newborns, as large as 97% of breastfeeding women use HM to overcome challenges with breastfeeding, and concerns are rising about the level of knowledge among healthcare providers on breastfeeding women’s HM use (Sim et al., 2013; Bettiol et al., 2018b; James et al., 2019; Zheng et al., 2020).

Thus, several studies have examined breastfeeding mothers’ HM use in various cultural settings (Bnouham, 2010; Sim et al., 2013; Aleandri et al., 2014; Bettiol et al., 2018b; James et al., 2019; Orabi et al., 2020; Zheng et al., 2020). However, limited data is available regarding the HM use in East African countries like Tanzania even though HM use is widely practiced to treat various physical conditions such as women’s complications as well as infectious and chronic diseases (Augustino and Gillah, 2005; Moshi et al., 2009; Amri and Kisangau, 2012; Kacholi and Amir, 2022). Consequently, breastfeeding mothers’ self-care practices must be examined to establish appropriate policy measures and clinical protocols to ensure the health of the mother and the child. Therefore, this study aims to examine the pattern and factors associated with HM use among breastfeeding mothers in Tanzania. Furthermore, this study emphasizes the importance of informing policymakers for developing a clinical guideline that could improve the self-care behaviors of women during breastfeeding.

## Materials and Methods

### Study Design

A descriptive cross-sectional study was conducted to identify HM use among breastfeeding mothers in Morogoro, Tanzania.

### Study Setting and Participants

The eligible participants included all breastfeeding women but are not limited to exclusive breastfeeding women between the ages of 18–49 years old with a child below 2 years who visited the reproductive and child health clinic at Uhuru health center in Morogoro, Tanzania. On average, approximately 160 pregnant women visit the Uhuru health center per month, and about 20% of those women visit the center for the reproductive and child health services. Exclusion criteria were those women attending the clinic for services apart from reproductive and child healthcare services and those who did not consent to participate in the survey.

### Study Size

The sample size determination formula, based on the confidence interval (CI) was used to calculate the required sample size: $n=z_{\alpha /2}^{2}\cdot \frac{pq}{d^{2}}$ . In this equation, n is the required sample size, Z_α/2_ is 1.96 with a confidence interval of 95%, d is the margin of error set on 0.05, p is the expected proportion based on an average prevalence of HM use among breastfeeding mothers (72.3%) (Sim et al., 2013; Aleandri et al., 2014), and q is the proportion of women that do not use HM during breastfeeding (1-p). The required sample size for adequate power was 308, and a total of 400 survey questionnaires were distributed assuming a 30% non-response rate.

### Data Collection

A face-to-face interview using a structured questionnaire was conducted from August 2017 to November 2017. The survey was conducted by three trained surveyors from Tanzania. All participants were informed about the confidentiality of the study and were given verbal information and instructions regarding the research. Participants who completed the IRB-approved consent form were able to participate in the study. A total of 400 questionnaires were distributed, 28 questionnaires were incomplete (response rate: 93.0%); therefore, the data of 372 respondents were included in the analysis.

### Survey Instrument

The questionnaire was first developed in English based on previous studies investigating HM use among breastfeeding women (Sim et al., 2013). In order to measure the content and face validity of the survey instrument, the questionnaire was reviewed by three experts (two researchers who were maternal and child health experts in Korea and one researcher from Uhuru health center, Tanzania). The questionnaire was then translated into Swahili, which was translated back into English by a third party to confirm its accuracy. The questionnaire was pilot-tested on a sample of 20 participants to evaluate the clarity and reliability of the questions.

The final version of the questionnaire consisted of three sections with 34 items, including multiple-choice and open-ended questions. The first section included eight questions on the health-related characteristics of the breastfeeding mothers, such as general health status (1 = very good, 5 = very poor), number of children, age of the youngest child, duration of breastfeeding of the youngest child, uptake of counseling on breastfeeding, and problems/difficulties experienced during breastfeeding. The second section included 18 questions regarding HM use (i.e., types and frequency of HM used, age of the child when HM was first used, indications and reasons for HM use, source of information and recommendation of HM, the experience of side effects, perceived safety and efficacy of HM, intention to recommend HM to other women, and disclosure and non-disclosure of HM use to health physicians and its reasons). Lastly, the final section consisted of eight questions on the participants’ sociodemographic characteristics such as age, level of education, occupation, number of family members in the household, household income, health insurance status, and marital status.

### Statistical Analysis

In this study, the collected data of 372 participants were analyzed using Statistical Package for Social Sciences (SPSS) version 24.0. The descriptive statistics were used to examine the sociodemographic characteristics of the participants (i.e., age, education level, family size, occupation, marital status, number of assets, health insurance status, perceived health status, number of children, exclusive breastfeeding, and the types of breastfeeding problems). A Chi-square test was used to analyze the differences in sociodemographic characteristics between the users of HM during breastfeeding and the non-users. Only the statistically significant variables (i.e., education level, number of children, and low breastmilk supply) from previous chi-square analysis were analyzed using multivariate logistic regression analysis to identify the potential predictors of HM use among breastfeeding mothers.

### Ethical Clearance

The study was approved by the Institutional Review Board on Human Subjects Research and Ethics Committees at Hanyang University Seoul, Korea (HYI-17-134-2). Formal permission was also obtained from Morogoro Municipal Council in Tanzania to carry out the study (E10/MMC-138/VOLIV/100).

## Results

### Sociodemographic Characteristics of Study Participants

The details of the sociodemographic characteristics of respondents are shown in Table 1 The mean age of breastfeeding mothers was 27.4 ± 5.3 years. Most participants were secondary school graduates (31.7%), or had a certificate/diploma and above (19.9%), were married (50.3%), and did not have health insurance (75.8%). Mothers had one (41.4%) or two (30.4%) children, and 54.8% of respondents exclusively breastfed their children. Among the respondents, 23.7% reported having problems with breastfeeding, and low breastmilk supply (14.8%) was the most commonly reported problem. More than half (53.8%) of the respondents have used at least one of the HM modalities listed during breastfeeding. Significant differences found between HM users and non-users were education level (*p* = 0.001), number of children (*p* = 0.008), having breastfeeding problem (*p* < 0.001), and low breastmilk supply (*p* < 0.001).

**TABLE 1:** Socio-demographic characteristics of participants (N =372).

Variables	Total		HM users	Non-users	*P*-value
	N=372 (%)		N=200 (%)	N=172 (%)	
Age (Mean± SD)	27.40 ± 5.33		27.30 ± 5.32	27.51 ± 5.35	—
≤ 24	119 (32.0)		67 (33.5)	52 (30.2)	0.707
25 ∼ 29	139 (37.4)		75 (37.5)	64 (37.2)	—
≥ 30	114 (30.6)		58 (29.0)	56 (32.6)	—
Education level	—		—	—	0.001
Primary education level and below	29 (7.8)		9 (4.5)	20 (11.6)	—
Primary education level	151 (40.6)		69 (34.5)	82 (47.7)	—
Secondary education level	118 (31.7)		74 (25.6)	44 (25.6)	—
Certificate/Diploma and above	74 (19.9)		48 (24.0)	26 (15.1)	—
Family Size	—		—	—	0.793
≤ 3	120 (32.3)		64 (32.0)	56 (32.6)	—
4 ∼ 5	175 (47.0)		92 (46.0)	83 (48.3)	—
≥ 6	77 (20.7)		44 (22.0)	33 (19.2)	—
Occupation	—		—	—	0.650
Employed	91 (24.5)		54 (27.0)	37 (21.5)	—
Street-vendor	113 (30.4)		59 (29.5)	54 (31.4)	—
Self-employed	75 (20.2)		40 (20.0)	35 (20.3)	—
Housewife	93 (25.0)		47 (23.5)	46 (26.7)	—
Marital status	—		—	—	0.863
Without spouse	63 (16.9)		32 (16.0)	31 (18.0)	—
Married	187 (50.3)		101 (50.5)	86 (50.0)	—
Cohabiting	122 (32.8)		67 (33.5)	55 (32.0)	—
Number of assets ^†^	—		—	—	0.171
≤ 3	139 (37.4)		69 (34.5)	70 (40.7)	—
4—6	141 (37.9)		74 (37.0)	67 (39.0)	—
≥ 7	92 (24.7)		57 (28.5)	35 (20.3)	—
Health insurance	—		—	—	0.882
No	282 (75.8)		151(75.5)	131(76.2)	—
Health insurance	90 (24.2)		49(24.5)	41(23.8)	—
Perceived health status	—		—	—	0.750
Excellent	101 (27.2)		53 (26.5)	48 (27.9)	—
Very good	240 (64.5)		132 (66.0)	108 (62.8)	—
Fair	31 (8.3)		15 (7.5)	16 (9.3)	—
Number of Children	—		—	—	0.008
1	154 (41.4)		96 (48.0)	58 (33.7)	—
2	113 (30.4)		49 (24.5)	64 (37.2)	—
3	105 (28.8)		55 (27.5)	50 (29.1)	—
Exclusive Breastfeeding	—		—	—	0.782
Yes	204 (54.8)		111 (55.5)	93 (54.1)	—
No	168 (45.2)		89 (44.5)	79 (45.9)	—
Breastfeeding problem	—		—	—	< 0.001
No	284 (76.3)		131 (65.5)	153 (89.0)	—
Yes	88 (23.7)		69 (34.5)	19 (11.9)	—
Types of breastfeeding problems	—	—	—	—	—
Low breastmilk supply	Yes	55 (14.8)	50 (25.0)	5 (2.9)	< 0.001
	No	317 (85.2)	150 (75.0)	167 (97.1)	—
Swollen breasts, sores on breasts, Cracked nipples, mastitis	Yes	30 (8.1)	21 (10.5)	9 (5.2)	0.063
	No	342 (91.9)	179 (89.5)	163 (94.8)	—
Breast engorgements, pain	Yes	6 (1.6)	4 (2.0)	2 (1.2)	0.523
	No	336 (98.4)	196 (98.0)	170 (98.8)	—

Note: HM, Herbal medicine.

a Type of assets were based on the Tanzania Bureau Statistics Survey 2012–2014 (Car, Bajaj, Motorcycle, Bicycle, Cart, Fridge, Electric or gas cooker, Television, Electric/charcoal iron, Cell phone, Radio, Plough, Charcoal / kerosene cooker, Livestock, Power tiller, House, Farm/Land).

b Columns do not add up to 100% due to the selection of multiple answers.

### Types of HM Modalities for Breastfeeding

As presented in Table 2, the most commonly used HM modality was *Piper nigrum* L. (80.0%), followed by *Cucurbita pepo* L. (18.0%) and *Arachis hypogaea* L. (12.0%).

**TABLE 2:** Types of HM used by breastfeeding mothers (N = 200).

Common name(s)^a^	Binomial name(s)	N (%)
Black pepper	*Piper nigrum* L	160 (80.0)
Pumpkin seeds	*Cucurbita pepo* L	36 (18.0)
Raw groundnuts	*Arachis hypogaea* L	23 (12.0)
Ginger	*Zingiber officinale* Roscoe	19 (10.0)
Lemon	*Citrus limon* (L.) Osbeck	12 (6.0)
Cinnamon	*Cinnamomum verum* J. Presl	12 (6.0)
Garlic	*Allium sativum* L	10 (5.0)
Raw cassava roots	*Manihot esculenta* Crantz	7 (4.0)
Lemongrass	*Cymbopogon citratus* Stapf	7 (4.0)
Aloe Vera	*Aloe vera* L	7 (4.0)
Clove	*Syzygium aromaticum* (L.) Merr. and L.M. Perry	5 (3.0)
Neem tree leaves	*Azadirachta indica* A. Juss	2 (1.0)

a Columns do not add up to 100% due to the selection of multiple answers.

### Source of Information on HM

The primary information sources of HM use were family members and friends (67.5%) and other breastfeeding mothers (31.5%), 26.5% from healthcare professionals, and 1.0% from herbalists (Table 3).

**TABLE 3:** Breastfeeding mother’s experience and patterns of HM use.

Variables	N	%
Conditions for HM use		
Low breastmilk supply	160	80.0
Swollen breasts, breast engorgement, sores, pain during breastfeeding	47	23.5
Body pain (backache, headache)	12	6.0
Any chronic illness (NCD’s)	11	5.5
Frequency of HM use		
Daily	179	89.5
Weekly	21	10.5
Experience side effects after using HM		
No	182	91.0
Yes	18	9.0
Sources of Information^a^		
Family member and friends	135	67.5
Other breastfeeding mother	63	31.5
Health professional	53	26.5
Herbalists	2	1.0
Internet	2	1.0
TV, radio and newspaper	2	1.0
Recommendation^a^		
Family member and friends	140	70.0
Other breastfeeding mother	64	32.0
Health professional	51	25.5
Herbalists	2	1.0
Reasons for using HM^a^ (N = 200)		
Herbal medicine can improve breastmilk production	184	92.0
Herbal medicine is cheap and easily accessible	44	22.0
Experience of using HM treatment in family	21	10.5
Herbal medicine have no side effects, nor chemicals	20	10.0
Herbal medicine can improve both my health and child health	15	7.5
Disclosure of HM use to physician		
Yes	55	27.5
No	145	72.5
Reason for non-disclosure^a^ (N = 145)		
Doctor did not ask me	94	64.8
I was afraid to tell the doctor	47	32.4
I thought it was not important to tell the doctor	7	4.8
There was not an important reason	5	3.4

a Columns do not add up to 100% due to the selection of multiple answers.

### Patterns of HM Use Among Participants

The experience and pattern of HM use during breastfeeding are presented in Table 3. The majority of HM users (89.5%) used the herbal products daily, and most women received recommendations on HM use from their family members and friends (70.0%). Among the HM users, 9.0% reported to have experienced side effects such as nausea, abdominal pain, and diarrhea. The most frequently reported reason for using HM during breastfeeding was the belief in improving low breastmilk supply (92.0%). A smaller proportion of HM users believed that HM does not cause side effects nor contains chemicals (10.0%). Among 200 participants using HM, 145 (72.5%) did not disclose their use to physicians, and the most common reasons for non-disclose were lack of physician inquiry (64.8%) and women’s fear of the doctor’s response (32.4%).

### Potential Predictors of HM Use During Breastfeeding

The findings from multivariate logistic regression analysis are presented in Table 4. An education level of certificate/diploma and above (OR: 7.447, CI: 2.492–22.252, *p* < 0.001), secondary education level (OR: 5.524, CI: 1.943–15.711, *p* = 0.001), primary education level (OR: 3.348, CI: 1.197–9.361, *p* = 0.021), and having low breast milk supply (OR: 13.758, CI: 5.022–37.689, *p* < 0.001) were associated with HM use during breastfeeding.

**TABLE 4:** Potential predictors of HM use among breastfeeding mothers using multivariate logistic regression analysis.

Variables		OR	95% CI	*p*-value
Educational level	Primary education level and below	1	Ref	—
	Primary education level	2.980	1.058–8.396	0.039
	Secondary education level	5.248	1.833–15.024	0.002
	Certificate/Diploma and above	8.325	2.745–25.242	<0.001
Number of Children	1	1	Ref	—
	2 or more	0.803	0.498–1.296	0.369
Low breastmilk supply	No	1	Ref	—
	Yes	15.526	5.564–43.327	<0.001
Asset holdings	No	1	Ref	—
Livestock				
	Yes	2.195	1.285–3.747	0.004

## Discussion

This study explored the prevalence and determinants of HM use among breastfeeding mothers in Tanzania, and our results revealed the high popularity of HM use among nursing mothers. As previous studies found, globally, 37.0–97.3% of mothers use HM to ease breastfeeding problems (Sim et al., 2013; Aleandri et al., 2014; Bettiol et al., 2018b; James et al., 2019; Zheng et al., 2020). Such variations in utilization rate can be attributed to differences in how HM is defined in each study, as well as the differences in the public acceptance and availability of HM due to cultural influences (Sim et al., 2013; James et al., 2019). Considering the low prevalence of exclusive breastfeeding and lack of appropriate breastfeeding knowledge among Tanzanian women (Hashim et al., 2016; Hasselberg et al., 2016; Kaaya et al., 2021) due to various individual, socio-cultural, and environmental barriers (Agho et al., 2019; Mundagowa et al., 2021), appropriate use of HM as galactagogues can potentially allow the mothers to engage in optimal breastfeeding practices and act as a safe alternative to synthetic galactagogues (Penagos-Tabares et al., 2014; Jyotsna and Sameet, 2020).

Similar to the broad range observed in the prevalence of HM use, wide varieties of HM were used by breastfeeding mothers in different countries. In Tanzania, the most commonly used herbal products were *P. nigrum* and *C. pepo*, whereas in Sierra Leone, *Cassia sieberiana* DC*.* and *Luffa acutangula* (L.) Roxb. were the most popular (James et al., 2019). *T. foenum-graecum* and *Z. officinale* use was the highest during lactation in Australia (Sim et al., 2013), while *Tetrapanax papyrifer* (Hook) K. Koch and *Vaccaria segetalis* (Neck.) Garcke consumption were most prevalent in Macau (Zheng et al., 2020). Such differences in preferred modalities can be due to different cultural traditions, as each traditional medicine is rooted in varying climate settings and religious backgrounds (Jaiswal and Williams, 2017; Tesfahuneygn and Gebreegziabher, 2019; Zhang and Dong, 2020). In addition, differences in the main indications for use reported among the studies may explain variations in HM modalities observed. For example, in our study, the most reported indication for HM was poor milk production, yet, in the Italian study, the main indications for HM use were dermatological conditions such as preventing stretch marks and preparing nipples for breastfeeding (Aleandri et al., 2014). Furthermore, among the Macau women, the primary indications for use were in relation to modulating the amount of breastmilk produced and managing breast and nipple pain (Zheng et al., 2020). These findings indicate that variations in the types of HM used can be expected depending on women’s cultural background and main indications for use (Forinash et al., 2012; Budzynska et al., 2013).

The procedures for preparing herbal remedies also vary by cultural background. *P. nigrum*, used by 80% of mothers in this study, is considered a medicinal plant in various cultures (Jamal et al., 2011; Takooree et al., 2019) and is specifically used to promote breastmilk production and postpartum care in India, Indonesia, and Morocco (Ramarao et al., 2000; Buragohain, 2008; Bnouham, 2010; Dandotiya et al., 2013; Chellappandian et al., 2014; Goyal, 2017; Muslichah et al., 2021). Depending on the cultural tradition, *P. nigrum*’s fruits are often mixed with other herbs (i.e., *Terminalia chebula* Retz., *Piper longum* L., *Zingiber officinale* Rosc., *Curcuma aeruginosa* Roxb., and *Piper betel* L.) and prepared as herbal concoctions to be consumed as galactagogue (Jyotsna and Sameet, 2020; Muslichah et al., 2021). Although not native to Africa, *P. nigrum* is also cultivated in East, Central, and West African regions and is often used as food condiments, insecticides, and cold-remedy (Kuete, 2017). It is also considered a galactagogue in places, and lactating mothers are recommended to consume a porridge with *P. nigrum* to promote breastmilk production (Hasselberg et al., 2016; Makwetta, 2021).

Nevertheless, despite women’s high dependency on HM to improve their breastfeeding due to the perception that HM is a natural and safe alternative to conventional medicine, HM’s clinical efficacy and safety as galactagogues still remain inconclusive and need further research (Sim et al., 2013; Zheng et al., 2020). For example, in the case of *P. nigrum*, its reputation as a lactogenic agent have received limited attention and relies on anecdotal evidence rather than clinical (Khare, 2007; Takooree et al., 2019). This may be because modalities such as *P. nigrum* are considered a food additive rather than a product with pharmacological value in many cultures, the importance of examining the effectiveness and potential dangers of such modalities can be neglected (Heinrich, 2016).

Although *P. nigrum* is better recognized for its anti-bacterial and anti-inflammatory effects in the existing literature, previous findings suggest its potential indirect effect as a galactagogue (Takooree et al., 2019). For instance, piperine, the active alkaloid contained in *P. nigrum*, improves the digestive capacity and enhances the bioavailability of drugs by increasing absorption activity in the intestines (Srinivasan, 2007; Goyal, 2017; Haq, et al., 2021) Piperine also possesses antioxidant properties which offer protection against oxidative stress by removing free radicals, reactive oxygen species, and chemical carcinogens (Srinivasan, 2007; Haq, et al., 2021). Managing postpartum oxidative stress is important as prolonged stress is involved in the pathogenesis of various maternal and neonatal diseases and can potentially alter the quality of breastmilk (Ozsurekci & Aykac, 2016; Kuramoto & Kitagawa, 2017). As consumption of balanced meals and proper nutrition is crucial for successful postpartum recovery and breastfeeding, piperine’s positive effect on the digestive tract, as well as the antioxidative effect may have helped the mothers to maintain good health and nutritional status to sustain optimal breastfeeding (Mistry & Williams, 2011; Goyal, 2017; Haq, et al., 2021). Nevertheless, as lactating mothers’ consumption of *P. nigrum* results in the transfer of piperine into the breastmilk, its potential effect on newborns warrants further investigation (N' Diaye et al., 2021).

Consumption of *C. pepo* was also prevalent among Tanzanian mothers. An animal study found that phytochemicals of *C. pepo* extract have a stimulatory effect on the pituitary gland, which stimulates prolactin secretion and increases the production of breast milk (Malgwi et al., 2013). However, further pharmacological research is required to ascertain their clinical efficacy in humans. As such, large-scale human clinical trials should be conducted for various traditional modalities to generate scientific evidence and demonstrate the safety and effectiveness of HM used among breastfeeding mothers (Jyotsna and Sameet, 2020).

In addition to the lack of scientific evidence to support HM’s effectiveness as galactagogues, the potential toxicity of HM due to heavy metal contamination is also a significant problem (Okem et al., 2014; Zhou et al., 2019; Luo et al., 2020). Because heavy metal components can pass through the breastmilk, inappropriate use of HM can expose infants to toxic substances (Palmieri et al., 2019). In fact, a study from Taiwan found that mothers’ consumption of Chinese herbal medicine was associated with the lead body burden in infants, posing potential health risks (Chien et al., 2006). The problems with contaminated HM have received considerable attention among academics and policymakers as HM is often obtained from unreliable sources such as local herbal shops and supermarkets; therefore, it is difficult to determine whether herbal products are manufactured and processed safely (Kunle et al., 2012; Sim et al., 2013; Aleandri et al., 2014). Thus, regulatory measures should be established to properly monitor the production of herbal products.

Lack of communication on HM use between the healthcare providers and nursing mothers is also a growing issue as poor communication can inhibit proper physician intervention when potential harm arises from inappropriate HM use (Foley et al., 2019). Congruent with previous findings, more than half of breastfeeding mothers in this study did not disclose their HM use to the doctors because the doctors did not ask and the women feared the doctors’ negative response (Foley et al., 2019; James et al., 2019). This implies that to prevent the potential risk of HM associated with inadequate patient-doctor communication, the physicians should actively inquire the nursing mothers about HM use and provide appropriate feedback to protect the health of the mothers and children.

Lastly, potential predictors of HM use identified in our sample include having low breastmilk supply and higher education attainment. Higher education level was frequently associated with the use of the non-conventional mode of care as people with higher education levels tend to have better health literacy, thus are more likely to practice patient autonomy and actively seek additional modes of care (Kemppainen et al., 2018; Fjær et al., 2020). Additionally, previous studies also found the age of the breastfeeding child, family income, ethnicity, previous use and positive attitudes towards non-conventional therapies, and the perceived accessibility of reliable information on HM use to be associated with HM use (Sim et al., 2013; Bettiol et al., 2018b; James et al., 2019; Zheng et al., 2020).

Several limitations can be found in this study. Because the study was conducted at a single health center, the views and opinions of study participants may not fully represent those of the other breastfeeding women. In addition, a recall bias can exist due to the retrospective design of the study. Lastly, cross-sectional data only provides information regarding the association between HM use and potential predictor variables. However, this is the first study to document the pattern of HM use among breastfeeding mothers in Tanzania and identify the factors that are significantly associated with HM use.

## Conclusion

There is a high prevalence of HM use among breastfeeding mothers in Tanzania to overcome various problems with breastfeeding. Appropriate HM use can be used as one of the strategies to achieve EBF as it can help nursing mothers from resource-limited countries to tackle common breastfeeding challenges and practice optimal feeding behavior. However, the effectiveness and safety of the most commonly used HM among nursing mothers rely on anecdotal evidence rather than clinical, and this awakens the need to evaluate HM’s safety and efficacy through pharmacological studies for scientific evidence. In addition, despite the popular use of HM among nursing mothers, no guideline has been established in clinical settings to promote open dialogues between the healthcare providers and mothers to ensure the safe use of HM. This puts emphasis on the development of public awareness program to encourage active patient-physician communication and facilitate proper HM use.

## Data Availability

The raw data supporting the conclusions of this article will be made available by the authors, without undue reservation.

## Abbreviations

HM, herbal medicine; WHO, world health organization; EBF, exclusive breastfeeding.
